# Comparative Chemical Profiles and Phytotoxic Activity of Essential Oils of Two Ecospecies of *Pulicaria undulata* (L.) C.A.Mey

**DOI:** 10.3390/plants10112366

**Published:** 2021-11-03

**Authors:** Ahmed M. Abd-ELGawad, Saud L. Al-Rowaily, Abdulaziz M. Assaeed, Yasser A. EI-Amier, Abd El-Nasser G. El Gendy, Elsayed Omer, Dakhil H. Al-Dosari, Giuliano Bonanomi, Hazem S. Kassem, Abdelsamed I. Elshamy

**Affiliations:** 1 Plant Production Department, College of Food and Agriculture Sciences, King Saud University, P.O. Box 2460, Riyadh 11451, Saudi Arabia; srowaily@ksu.edu.sa (S.L.A.-R.); assaeed@ksu.edu.sa (A.M.A.); dadosari@gmail.com (D.H.A.-D.); 2 Department of Botany, Faculty of Science, Mansoura University, Mansoura 35516, Egypt; yasran@mans.edu.eg; 3 Medicinal and Aromatic Plants Research Department, National Research Centre, 33 El Bohouth St., Dokki, Giza 12622, Egypt; aggundy_5@yahoo.com (A.E.-N.G.E.G.); sayedomer2001@yahoo.com (E.O.); 4 Department of Agriculture, University of Naples Federico II, Portici, 80055 Naples, Italy; giuliano.bonanomi@unina.it; 5 Department of Agricultural Extension and Rural Society, College of Food and Agriculture Sciences, King Saud University, Riyadh 11451, Saudi Arabia; hskassem@ksu.edu.sa; 6 Department of Natural Compounds Chemistry, National Research Centre, 33 El Bohouth St., Dokki, Giza 12622, Egypt; elshamynrc@yahoo.com

**Keywords:** allelopathy, *Pulicaria crispa*, chemometric analysis, chemotype, Asteraceae

## Abstract

The Asteraceae (Compositae) family is one of the largest angiosperm families that has a large number of aromatic species. *Pulicaria undulata* is a well-known medicinal plant that is used in the treatment of various diseases due to its essential oil (EO). The EO of both Saudi and Egyptian ecospecies were extracted via hydrodistillation, and the chemical compounds were identified by GC–MS analysis. The composition of the EOs of Saudi and Egyptian ecospecies, as well as other reported ecospecies, were chemometrically analyzed. Additionally, the phytotoxic activity of the extracted EOs was tested against the weeds *Dactyloctenium aegyptium* and *Bidens pilosa*. In total, 80 compounds were identified from both ecospecies, of which 61 were Saudi ecospecies, with a preponderance of *β*-pinene, isoshyobunone, 6-epi-shyobunol, *α*-pinene, and *α*-terpinolene. However, the Egyptian ecospecies attained a lower number (34 compounds), with spathulenol, hexahydrofarnesyl acetone, *α*-bisabolol, and τ--cadinol as the main compounds. The chemometric analysis revealed that the studied ecospecies and other reported species were different in their composition. This variation could be attributed to the difference in the environmental and climatic conditions. The EO of the Egyptian ecospecies showed more phytotoxic activity against *D. aegyptium* and *B. pilosa* than the Saudi ecospecies. This variation might be ascribed to the difference in their major constituents. Therefore, further study is recommended for the characterization of authentic materials of these compounds as allelochemicals against various weeds, either singular or in combination.

## 1. Introduction

Taxa belonging to *Pulicaria* (Asteraceae Family) are widely distributed in Asia, Africa, and Europe. These plants are considered very important medicinal plants due to their traditional applications around the world, in addition to the presence of interesting metabolites comprising mono-, sesqui-, and diterpenoids, as well as phenolic and flavonoids [1,2,3,4].

The Egyptian widespread desert plant, *Pulicaria undulata* (L.) (syn. *Pulicaria crispa* (Forssk.) Benth et Hook), was documented as a very important traditional plant for the treatment of diabetes, abscesses, cardiac and skin diseases, and chills [5]. In Egypt, this plant was used as a herbal tea for inflammation treatment, in addition to insect repellent [4]. 

Numerous pharmaceutical activities were described for different extracts and ingredients of this plant such as antioxidant [6,7,8], neuroprotective [9], antiulcer [10], antiacetylcholinesterase [8], anticancer [11], and *α*-glucosidase inhibitory activity [12]. These biological activities of *P. undulata* were ascribed to different classes of identified chemical compounds such as terpenes [4,12,13,14,15], flavonoids [7,16], and sterols [11]. The essential oil (EO) of *P. undulata* exhibited various potent biological activities such as antiproliferative, antioxidant [6,15], anticancer [8], antibacterial [13], and cytotoxic [12].

Many documents have been published concerning the chemical characterization EOs of different ecospecies of *P. undulata* from different countries such as Sudan [6,17], Iran [15,18,19], Algeria [14], Yemen [13], and Egypt [8,16,20]. However, by comparing all these ecospecies, there was evidence that their EOs were different either in quality or quantity. This deduced that the biosynthesis of the natural metabolites including EOs in the plant kingdom is correlated with environmental and climatic conditions, in addition to genetic variability [21,22,23]. The present study aimed to analyze and compare the EO profiles of two ecospecies of *P. undulata* growing in Saudi Arabia and Egypt, assess phytotoxicity against the noxious weeds *Dactyloctenium aegyptium* and *Bidens* *pilosa,* as well as holistically categorize their EOs with other reported ecospecies using chemometric tools.

## 2. Results and Discussion

### 2.1. Yields and Chemical Constituents of P. undulata EOs 

The aerial parts of Saudi and Egyptian *P. undulata* (150 g each) were subjected separately to the hydrodistillation for 3 h in Clevenger-type apparatus, provided pale yellow oil with an average yield of 0.43% and 0.36% (*v*/*w*), respectively. The yields of EOs in our study were comparable to those reported from other Egyptian ecospecies (0.23–0.60%) [9,16,20]. However, the yield of the present *P. undulata* ecospecies was lower than that reported in previous studies for other ecospecies such as Yemeni (2.10%) [13], Iranian (0.50–1.34%) [15,19,24], Sudanian (1.40–2.50%) [6,17], and Algerian ecospecies (1.20%) [14]. These variations in the yield of the EOs might be attributed to the difference in the geographical region, in addition to the environmental conditions such as soil, climate, as well as genetic pool [22,25,26,27].

**Figure 1:**
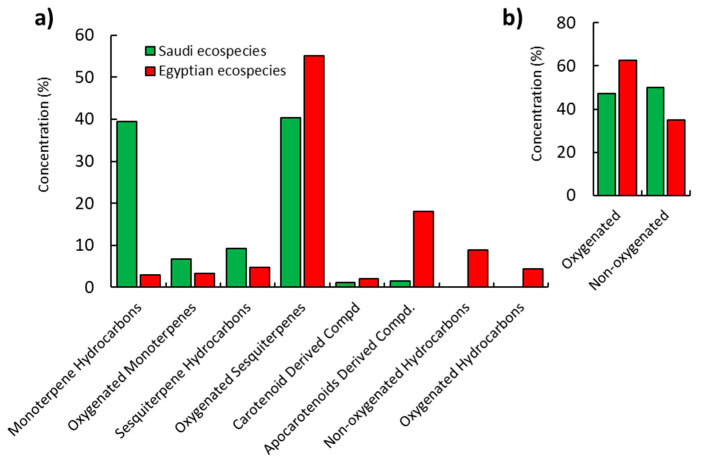
Classification of the chemical compounds of *Pulicaria undulata* EOs of Saudi and Egyptian ecospecies. (**a**) various classes and (**b**) oxygenated and non-oxygenated compounds.

In total, 80 compounds were characterized depending upon GC–MS analysis of the two EOs of *P. undulata* including 61 and 34 compounds from Saudi and Egyptian ecospecies, respectively. The identified constituents were classified into eight classes—namely, (i) monoterpene hydrocarbons, (ii) oxygenated monoterpenes, (iii) sesquiterpene hydrocarbons, (iv) oxygenated sesquiterpenes, (v) carotenoid-derived compounds, (vi) apocarotenoid-derived compounds, (vii) nonoxygenated hydrocarbons, and (viii) oxygenated hydrocarbons, (Figure 1a). Oxygenated sesquiterpenes were the most represented class—they represented 55.03% and 40.34% of the total oil of the Egyptian and Saudi ecospecies, respectively. Monoterpenes were determined with a high content (39.46%) of the EO of Saudi ecospecies, while it represented a minor class in Egyptian ecospecies (6.70%). Additionally, hydrocarbons represented 13.32% of the total EO content of the Egyptian eco-sample, while completely absent in the Saudi plant sample. Overall, the Egyptian ecospecies had oxygenated compounds as the main elements, while non-oxygenated compounds were represented as the main constituents of Saudi ecospecies (Figure 1b). The identified compounds, accounting for 97.22% and 97.61%, respectively, of overall EO mass, in addition to their retention times, and literature and experimental Kovats indices are presented in Table 1.

The analysis of the data revealed that the EOs of the two plant samples were very rich with terpenoids, with respective concentrations of 95.78% and 66.17% in addition to carotenoids (2.77% and 20.15%, respectively). The variations in the quantitative and qualitative analysis of EOs of the two plant samples were attributed directly to the environmental and climate variations between the Saudi and Egyptian environments [28,29].

More in-depth data indicated that the EO of the Saudi *P. undulata* contained mainly terpenoids, including almost equal concentrations of mono (46.27%) and sesquiterpenes (49.51%) with traces of carotenoids and a complete absence of diterpenoids and hydrocarbons. In comparison, the chemical characterization of the EO of the Egyptian plant showed that terpenoids were the major compounds, including minor elements of monoterpenes (6.40%) and abundance of sesquiterpenes (59.77%), as well as a high concentration of carotenoids. Similarly, the EO of the Egyptian plant was characterized by the complete absence of diterpenes and the presence of a remarkable concentration of hydrocarbons. The sesquiterpenes were found as major constituents of the EOs of both ecospecies (Saudi and Egyptian); this result was different than those reported for Yemeni leaves (2.1%) [13], Iranian aerial parts (0.5%) [15], and Egyptian aerial parts (0.6%) [20] of *P. undulata*. The sesquiterpenes in the EOs of Saudi and Egyptian ecospecies were categorized as sesquiterpene hydrocarbons (9.17% and 4.74%), and oxygenated sesquiterpenes (40.34% and 55.03%). Isoshyobunone (7.67%), 6-epi-Shyobunol (6.51%), spathulenol (3.43%), and *trans*-nerolidol (3.33%) represented the main oxygenated sesquiterpene of EO of the Saudi plant. In comparison, spathulenol (30.86%), *α*-bisabolol (6.34%), 4,4-dimethyl-tetracyclo[6.3.2.0(2,5).0(1,8)]tridecan-9-ol (4.68%), and τ--cadinol (3.65%) were found to be the abundant oxygenated sesquiterpenes of EO of Egyptian ecospecies. Most of the studied ecospecies of *P. undulata* have been described as non-rich of sesquiterpene [13,15,20]. However, EOs of other *Pulicaria* species such as *P. somalensis* [1], *P. dysenterica* [30], and *P. gnaphalodes* [31] were reported as rich in sesquiterpene.

Numerous *Pulicaria* plants were described to have spathulenol as minor and/or major compounds of their EOs such as *P. somalensis* [1] and *P. stephanocarpa* [32]. *α*-Bisabolol was detected as the main sesquiterpene in EOs derived from some *Pulicaria* species such as *P. somalensis* [1], *P. dysenterica* [30], and *P. gnaphalodes* [31]. Moreover, the major sesquiterpene, cadinol, in this study has been described as a major component in EO derived from aerial parts of the *P.* *undulata* collected from the Algerian Sahara [14], while it was reported as minor or trace element in other ecospecies.

Monoterpenes were reported as the main constituents of several *Pulicaria* ecospecies [13,33]. Saudi *P.* *undulata* was found to be in harmony with the reported documents where the monoterpenes represented around half of the total oil (46.27%) including hydrocarbons (39.46%) and oxygenated (6.81%) forms of monoterpene. However, the monoterpenes were identified as trace elements (6.40%) in the EO of Egyptian plants, including traces of non-oxygenated and oxygenated forms, with respective relative concentrations of 3.04% and 3.36%. In the EO from the Saudi sample, *β*-pinene (21.14%), *α*-pinene (5.08%), and *α*-terpinolene (3.84%) were assigned as the main monoterpene hydrocarbons, while myrtenyl acetate (2.50%) and terpinen-4-ol (1.20%) were characterized as main oxygenated monoterpenes. Only four monoterpenes were identified from overall compounds of EO of Egyptian ecospecies. *β*-Pinene (1.32%) was assigned as the main monoterpene hydrocarbons, and carvotanacetone (3.36%) was the only identified oxygenated one. Carvotanacetone was stated as the main monoterpene of *P.* *undulata* collected from Yemen [13] and from the Egyptian Western Desert region [8,20]. The present data revealed that the variations in the components in EO of Egyptian and Saudi samples might be attributed to the variations in collection areas, in addition to the environmental conditions such as soil, climate, as well as their genetic pool [23]. The abundance of pinene and myrtenyl derivatives, *α*-terpinolene, terpinen-4-ol was in complete harmony with the data reported from the Iranian *P.* *undulata* [15,18].

Carotenoid-derived compounds were represented as trace constituents in the EO of the Saudi ecospecies, with a concentration of 2.77%, comprising carotenoids (1.26%) and apocarotenoid-derived compounds (1.51%). Hexahydrofarnesyl acetone was found as the main component in all characterized carotenoid-derived compounds. By contrast, carotenoid-derived compounds derived from the Egyptian EO sample were characterized by high concentration (20.12%), representing carotenoid-derived compounds (2.03%) and apocarotenoid-derived compounds (18.12%). Additionally, hexahydrofarnesyl acetone represented the predominated compound in all overall carotenoid-derived compounds. Hexahydrofarnesyl acetone is a common apocarotenoid-derived compound in EOs derived from the plant kingdom such as *Hildegardia barteri* [34], *Stachys tmolea* [35], and *Bassia muricata* [36].

The hydrocarbons represented 13.32% of the total identified oil of the Egyptian plant involved non-oxygenated (8.98%) and oxygenated (4.34%) forms. *n*-nonadecane (1.57%) and *n*-nonacosane (1.49%) were identified as the majors of non-oxygenated hydrocarbons, while *cis*-9-hexadecenoic acid (3.73%) represented the main oxygenated hydrocarbons. Hydrocarbons were completely absent from the EO of the Saudi plant, and this result was found in agreement with Iranian *P. undulata* [15,18]. 

### 2.2. Chemometric Analysis of the EOs of Pulicaria Ecospecies 

**Figure 2:**
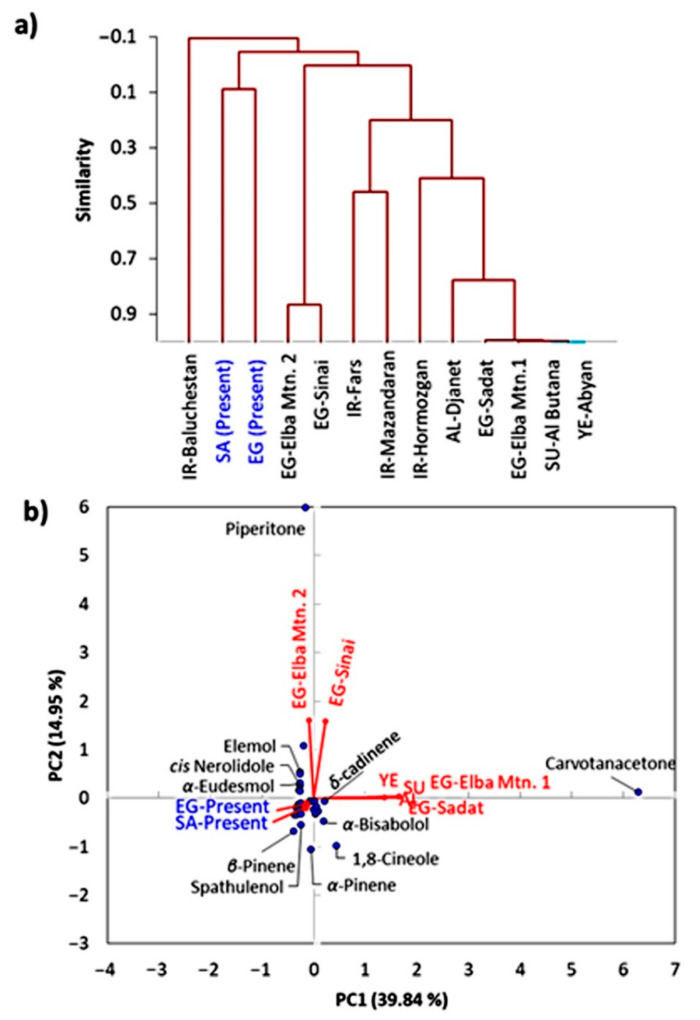
Chemometric analysis of the essential oil of different *Pulicaria undulata* ecospecies: (**a**) agglomerative hierarchical clustering (AHC) and (**b**) principal components analysis (PCA). SA: Suadi, EG: Egyptian, IR: Iranian, AL: Algerian, SU: Sudanian, and YE: Yemeni. The blue color represents the present samples.

The application of the EOs profiles of the 2 ecotypes of *P. undulata* and the other 11 ecotypes were subjected to principal component multivariate data analysis (PCA) and agglomerative hierarchical clustering (AHC). The cluster analysis revealed that the EOs could be categorized into four clusters. Cluster-I consisted of the Iran–Baluchestan ecotype, while the EOs of the presently studied ecospecies (Saudi and Egyptian) were grouped as cluster-II. Further, the Egypt–Elba Mountain-2 and Egypt–Sinai ecospecies showed a close correlation, and therefore, they were grouped as cluster-III. Finally, cluster-IV contained Iranian (Iran–Baluchestan, Iran–Fars, and Iran–Hormozgan samples), Algerian, Sudanian, Yemeni, Egyptian (Elba Mountain-2, and Sadat) ecospecies (Figure 2a).

The PCA score plot showed the distant separation of Egypt–Elba Mountain-2 and Egypt–Sinai ecospecies in the PC2, while Egypt–Elba Mountain-2, Egypt Sadat, Yemeni, Algerian, and Sudanian ecospecies were distantly distributed along the right side of the PC1 (Figure 2b). Conversely, the present samples (Saudi and Egyptian) as well as Iranian and Algerian were clustered together in the center of the PCA and had positive score values. In addition, the examination of the loading plot showed that piperitone was the most correlated/abundant compound in Egypt–Elba Mountain-2 and Egypt–Sinai ecospecies. However, carvotanacetone showed an abundance in Egypt–Elba Mountain-2, Egypt Sadat, Yemeni, Algerian, and Sudanian ecospecies. The detected variation among different ecospecies could be ascribed to the effect of climatic and environmental conditions, as well as the genetic characteristics [22,25,26,37].

### 2.3. Phytotoxic Activity of P. undulata EOs

**Figure 3:**
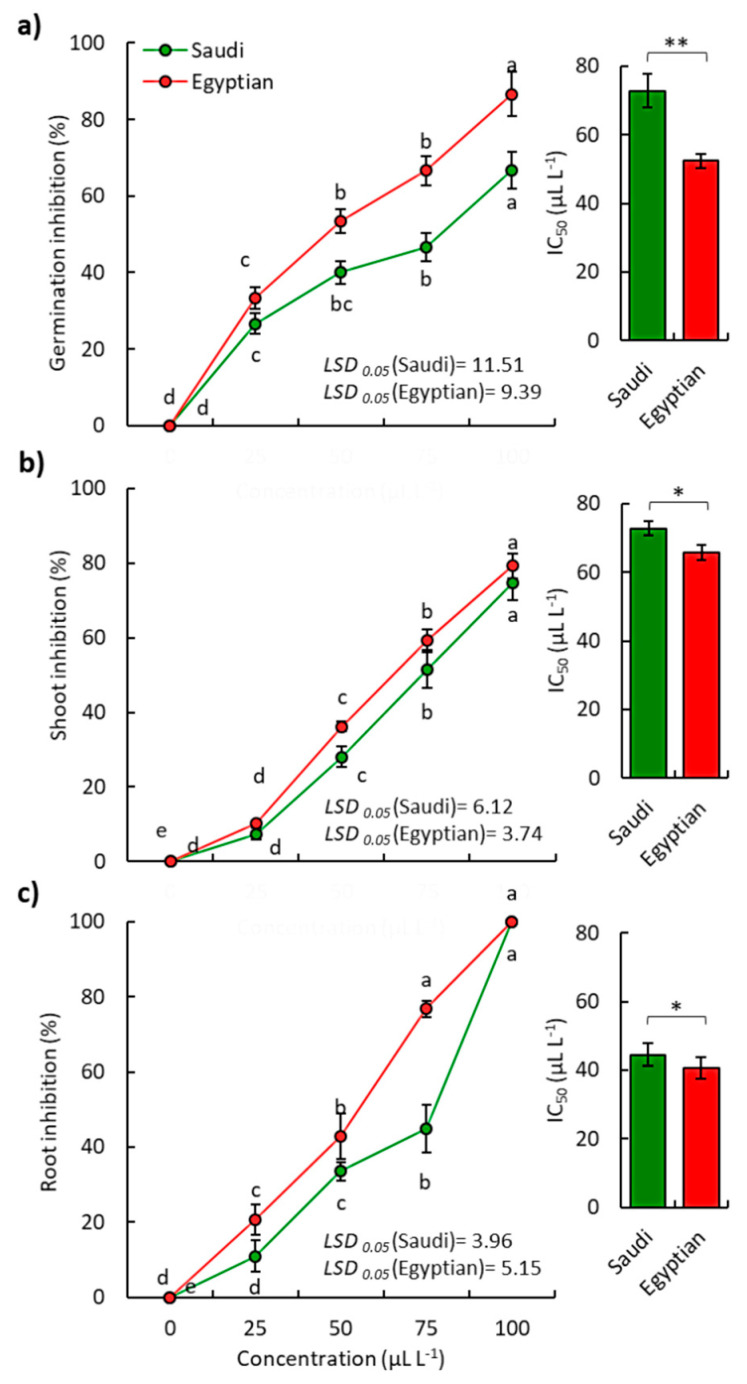
Phytotoxic effect of the EOs extracted from the aerial parts of both Saudi and Egyptian ecotypes of *P. undulata* on the (**a**) germination of seeds, (**b**) shoot growth, and (**c**) root growth of the weed *Bidens pilosa*. Different letters on each line mean significant differences (one-way randomized blocks ANOVA). Data are mean value (*n = 3*), and the bars represent the standard error. * *p* < 0.05, ** *p* < 0.01.

The EOs of both Saudi and Egyptian ecotypes of *P. undulata* showed significant phytotoxic activity against seed germination and seedling growth of the noxious weed *B. pilosa* (Figure 3). At the highest concentration (100 µL L^−1^), EOs of Saudi ecospecies showed inhibition of germination, shoot growth, and root growth of *B. pilosa* by 66.67%, 74.59%, and 83.47%, respectively, while the Egyptian species showed inhibition values of 86.67%, 79.23%, and 94.17%, respectively (Figure 3). Based on the IC_50_, the Saudi ecospecies showed IC_50_ values of 72.83, 72.84, and 44.55 µL L^−1^ regrading germination, shoot growth, and root growth of *B. pilosa*, respectively. However, the Egyptian ecospecies showed IC_50_ values of 42.42, 65.71, and 40.70 µL L^−1^, respectively (Figure 3).

It was evident that the Egyptian ecospecies were more effective against *B. pilosa* than Saudi ecospecies, which could be ascribed to the variation in the quality and quantity of the chemical composition of the EO. In this study, the Egyptian ecospecies were richer in oxygenated compounds than the Saudi ones. EOs rich in oxygenated compounds have been reported to possess more activity [38,39,40,41]. The phytotoxic activity of the EO from Egyptian ecospecies might be attributed to its major compounds such as spathulenol, hexahydrofarnesyl acetone, *α*-bisabolol, and τ--cadinol. Additionally, the Saudi ecospecies had *β*-pinene, isoshyobunone, 6-epi-shyobunol, *α*-pinene, and *α*-terpinolene as major compounds. Moreover, τ--cadinol was identified as a major compound in the EO of *Cullen plicata*, where it showed strong phytotoxic activity against *B. pilosa* and *Urospermum picroides* [38]. Additionally, τ--cadinol was reported in a high concentration of the EO of *Rhynchosia minima*, which showed significant allelopathic activity against *Dactyloctenium aegyptium* and *Rumex dentatus* [42]. However, *α*-bisabolol, as a major compound of the Egyptian ecospecies in the present study, has not been reported to possess phytotoxicity; therefore, further study is recommended for its characterization as an allelochemical compound.

In the Egyptian ecospecies, the major compound, spathulenol (30.86%), has also been reported as major compounds of EOs with substantial phytotoxic activity such as *Launaea mucronata* [26], *Xanthium strumarium* [37], *Eucalyptus camaldulensis* [43], *Teucrium arduini* [44], and *Symphyotrichum squamatum* [25]. Moreover, hexahydrofarnesyl acetone (18.12%), was determined in a high concentration of the EO, which exhibited strong phytotoxicity such as *Heliotropium curassavicum* [23], *Launaea nudicaulis*, *Launaea mucronata* [26], and *Bassia muricata* [36].

Otherwise, the main compound in the EO of Saudi ecospecies, *β*-pinene (21.14%), has been reported as the main compound of EOs of various plants that have exhibited phytotoxic activity such as *Schinus terebinthifolius* [45], *Symphyotrichum squamatum* [25], *Pinus brutia*, *Pinus pinea* [46], *Lavandula angustifolia* [44], and *Heterothalamus psiadioides* [47]. The other major compounds of the Saudi ecospecies have also been reported in EOs with significant phytotoxicity [1,46,48]. Additionally, the present data showed that the roots were more sensitive to the EO than shoots since roots were directly exposed to the EO. Moreover, root cells have more permeability than the cells of the shoot [22,38]. 

**Figure 4:**
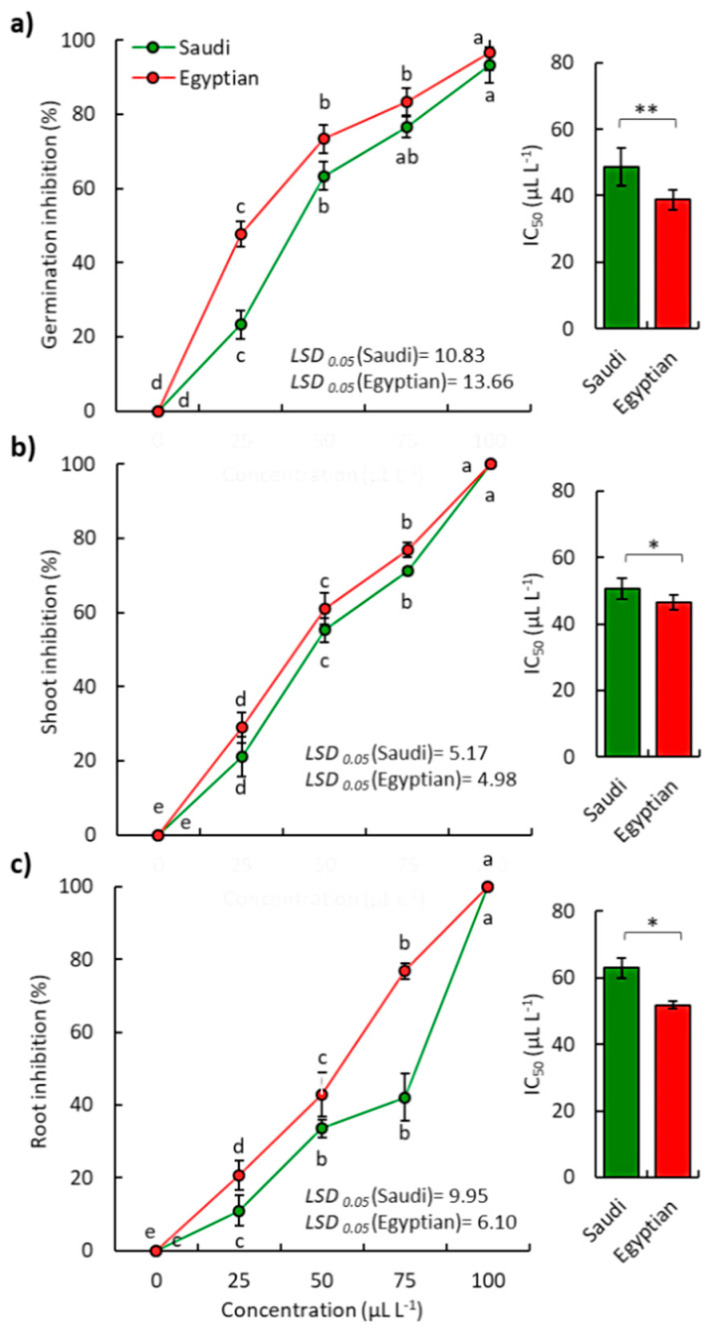
Phytotoxic effect of the EOs extracted from the aerial parts of both Saudi and Egyptian ecotypes of *P. undulata* on the (**a**) germination of seeds, (**b**) shoot growth, and (**c**) root growth of the weed *Dactyloctenium aegyptium*. Different letters on each line mean significant differences (one-way randomized blocks ANOVA). Data are mean value (*n = 3*) and the bars represent the standard error. * *p* < 0.05, ** *p* < 0.01.

Results also indicated that the EOs of Saudi and Egyptian ecospecies showed more inhibitory activity against the weed *D. aegyptium* than *B. pilosa* (Figure 4). 

At the highest concentration of the Saudi EOs (100 µL L^−1^), the *D. aegyptium* seedling growth was completely inhibited. However, the germination was reduced by 93.33%, while the Egyptian ecospecies showed 96.67%. Based on the IC_50_ values, the EO of the Saudi ecospecies showed IC_50_ values of 48.61, 50.49, and 62.92 µL L^−1^ for germination, shoot growth, and root growth of *D. aegyptium*, respectively, while the Egyptian ecospecies attained IC_50_ values of 38.84, 46.59, and 51.87 µL L^−1^, respectively. 

## 3. Materials and Methods

### 3.1. Plant Samples Collection and Preparation

The aerial parts of Saudi *P. undulata* were collected from the Wadi Alsahbaa, Alkharj, Riyadh region (24°16′34.1″ N 47°56′11.3″ E), while the Egyptian sample was collected from Wadi Hagoul, the Eastern Desert, Egypt (30°00′38.2″ N 32°05′35.5″ E), during spring of 2019. The specimens were authenticated according to Tackholm [49] and Boulos [50]. Voucher specimens were prepared and deposited in the herbarium of the Department of Botany, Faculty of Science, Mansoura University with No. Mans.001162117 and Mans.001162118. 

The samples were collected from two populations of *P. undulata* in separate plastic bags and immediately transferred to the lab. The samples were dried in a shaded place at room temperature (25 ± 3 °C) for 7 days, crushed into powder using a grinder (IKA^®^ MF 10 Basic Microfine Grinder Drive, Breisgau, Germany) at a dimension of 3.0 mm, and packed in paper bags.

### 3.2. EOs Extraction, GC–MS Analysis, and Chemical Compounds Identification

About 150 g of the prepared samples of *P. undulata* were extracted with hydrodistillation via a Clevenger-type apparatus for 3 h. The oils were collected, water was removed using 0.5 g of anhydrous sodium sulfate, and stored in glass vials in the fridge (−4 °C) till further analysis [29]. Two samples of the plant were extracted by the same protocol afforded two samples of EOs. The two extracted EOs were analyzed via gas chromatography–mass spectrometry (GC-MS) at the National Research Center, Giza, Egypt, as described in our previously documented work [25,26,48,51]. Briefly, the apparatus has TRACE GC Ultra Gas Chromatographs (THERMO Scientific™ Corporate, Waltham, MA, USA), together with Thermo Scientific ISQ™ EC single quadrupole mass spectrometer. The GC–MS system is equipped with a TR-5 MS column (0.25 µm film thickness, 30 m × 0.32 mm internal diameter). Helium was used as a carrier gas at a flow rate of 1.0 mL min^−1^, with a divided ratio of 1:10. The temperature program was 60 °C for 1 min, rising by 4.0 °C min^−1^ to 240 °C, and held for 1 min. An aliquot of 1 µL of the EO sample in hexane was injected at a ratio of 1:10 (*v*/*v*), and the detector and injector were adjusted at 210 °C. Mass spectra were recorded by electron ionization (EI) at 70 eV, using a spectral range of *m*/*z* 40–450. The chemical compounds identification was accomplished by Automated Mass spectral Deconvolution and Identification (AMDIS) software, as well as Wiley Spectral Library collection, NIST Library database (Gaithersburg, MD, USA; Wiley, Hoboken, NJ, USA), which were used for retention indices relative to n-alkanes (C_8_–C_22_), or appraisal of the mass spectrum with authentic standards.

### 3.3. Phytotoxic Activity Estimation of the EOs

The extracted EOs were tested for their phytotoxicity against two noxious weeds *Dactyloctenium aegyptium* and *Bidens pilosa*. The seeds of *D. aegyptium* were collected from cultivated fields near the Mediterranean coast, at Gamasa City, northern Egypt (31°27′03.9″ N 31°27′44.8″ E), while the seeds of *B. pilosa* were collected from a garden in Mansoura University campus, Mansoura, Egypt (31°02′40.2″ N 31°21′18.4″ E). The homogenous and ripe seeds were selected, sterilized with 0.3% sodium hypochlorite, rinsed with distilled and sterilized water, dried, and stored in sterilized vials.

The phytotoxicity experiments were conducted in vitro following the methodology described by Abd El-Gawad et al. [38]. In brief, 20 seeds of the weed were transferred to a Petri plate lined with Whatman No. 1 filter paper wetted with 4 mL of each concentration of the EOs (25, 50, 75, and 100 µL L^−1^). Different concentrations of the EOs were prepared using 1% Tween^®^ 80 (Sigma-Aldrich, Darmstadt, Germany) as an emulsifier. The plates were sealed with Parafilm^®^ tape and incubated in a growth chamber adjusted with a temperature of 25 °C and light/dark cycle of 12/12 h. Besides, Tween^®^ 80 was used as a control treatment. After seven days of incubation, the germinated seeds were counted and the length of shoots and roots of the seedlings were measured. The inhibition of germination and seedling growth were calculated based on the following equation:

$$\mathrm{Inhibition}\;\%=100\times \frac{\left(\mathrm{Length}/{\mathrm{Number}}_{\mathrm{Control}}-\mathrm{Length}/{\mathrm{Number}}_{\mathrm{Treatment}}\right)}{\left(\mathrm{Length}/{\mathrm{Number}}_{\mathrm{Control}}\right)}$$

The IC_50_ (the concentration of the EO required to reduce the germination or growth by 50%) was calculated using MS-Excel.

### 3.4. Data Analysis

The experiment of phytotoxicity was repeated three times with three replications. The data of the inhibition were subjected to one-way ANOVA, followed by Duncan’s test using CoStat program (version 6.311, CoHort Software, Monterey, CA, USA), while the IC_50_ values were subjected to a two-tailed *t*-test using MS-EXCEL. To make a holistic categorization of the EOs of the two studied ecospecies (Saudi and Egyptian) and other reported ecospecies (Algerian, Egyptian, Iranian, Sudanian, and Yemeni), we constructed a data matrix of the 30 major chemical compounds, with concentration > 3%, from 11 ecospecies. The matrix was subjected to Principal component multivariate data analysis (PCA) and agglomerative hierarchical clustering (AHC) using the XLSTAT Statistical Software package (version 2018, Addinsoft Inc., New York, NY, USA).

## 4. Conclusions

The EO composition of the Saudi and Egyptian ecospecies of *P. undulata* showed substantial variation in both quantity and quality. The Saudi ecospecies had 61 compounds, with *β*-pinene, isoshyobunone, 6-epi-shyobunol, *α*-pinene, and *α*-terpinolene as major compounds, while the EO of the Egyptian ecospecies attained a lower number (34 compounds), with spathulenol, hexahydrofarnesyl acetone, *α*-bisabolol, and τ--cadinol as main compounds. This variation could be attributed to the difference in the environmental and climatic conditions. The EO of the Egyptian ecospecies showed more phytotoxic activity against *D. aegyptium* than *B. pilosa*, as well as more phytotoxic, compared with the Saudi ecospecies. This variation might be ascribed to the difference in their major constituents. Therefore, further study is recommended for the characterization of authentic materials of these compounds as allelochemicals against various weeds, either singular or in combination.

## Figures and Tables

**Table 1:** Chemical constituents of the EOs of the aerial parts of Saudi and Egyptian ecospecies of *Pulicaria undulata*.

No.	Rt ^a^	Relative Conc. (%)		KI		Compound Name	Identification
		SA ^b^	EG ^c^	Lit. ^d^	Exp. ^e^		
Monoterpene Hydrocarbons							
1	4.05	0.14 ± 0.01	-----	931	931	*α*-Thujene	KI and MS
2	4.20	5.08 ± 0.06	0.91 ± 0.01	939	940	*α*-Pinene	KI and MS
3	4.58	0.15 ± 0.02	-----	953	951	Camphene	KI and MS
4	5.07	0.76 ± 0.04	-----	976	975	Sabinene	KI and MS
5	5.21	21.14 ± 0.12	1.32 ± 0.05	980	980	*β*-Pinene	KI and MS
6	6.19	0.65 ± 0.01	-----	1031	1030	Limonene	KI and MS
7	6.50	7.70 ± 0.08	-----	1064	1063	*γ*-Terpinene	KI and MS
8	8.09	3.84 ± 0.05	0.81 ± 0.03	1088	1086	*α*-Terpinolene	KI and MS
Oxygenated Monoterpenes							
9	5.50	0.46 ± 0.03	-----	991	990	Dehydro-1,8-cineole	KI and MS
10	5.92	0.22 ± 0.01	-----	1005	1005	*α*-Phellandrene	KI and MS
11	6.01	0.13 ± 0.01	-----	1129	1129	*p*-2-Menthen-1-ol	KI and MS
12	9.45	0.27 ± 0.02	-----	1131	1132	*trans*-*p*-Mentha-2,8-dienol	KI and MS
13	9.96	0.15 ± 0.01	-----	1137	1138	*β*-Nopinone	KI and MS
14	10.05	0.27 ± 0.02	-----	1139	1139	Pinocarveol	KI and MS
15	10.23	0.21 ± 0.01	-----	1140	1140	*cis*-Verbenol	KI and MS
16	10.72	0.27 ± 0.03	-----	1143	1145	Camphor	KI and MS
17	10.99	0.28 ± 0.02	-----	1162	1161	Pinocarvone	KI and MS
18	11.28	0.28 ± 0.01	-----	1165	1167	*endo*-Borneol	KI and MS
19	11.82	1.20 ± 0.04	-----	1177	1179	Terpinen-4-ol	KI and MS
20	13.04	0.20 ± 0.02	-----	1194	1193	Myrtenal	KI and MS
21	13.51	0.11 ± 0.01	-----	1228	1229	*α*-Citronellol	KI and MS
22	16.79	0.14 ± 0.01	-----	1321	1319	Isopulegol acetate	KI and MS
23	17.33	0.48 ± 0.01	-----	1354	1356	Citronellyl acetate	KI and MS
24	17.84	0.26 ± 0.01	3.36 ± 0.07	1258	1259	Carvotanacetone	KI and MS
25	20.21	2.50 ± 0.05	-----	1326	1326	Myrtenyl acetate	KI and MS
Sesquiterpene Hydrocarbons							
26	16.34	0.58 ± 0.01	-----	1377	1375	Berkheyaradulen	KI and MS
27	17.48	3.63 ± 0.04	1.17 ± 0.06	1409	1410	*α*-Gurjunene	KI and MS
28	17.70	0.62 ± 0.03	-----	1418	1418	*trans*-Caryophyllene	KI and MS
29	18.47	0.78 ± 0.02	-----	1439	1437	*α*-Guaiene	KI and MS
30	18.69	0.88 ± 0.04	0.81 ± 0.01	1455	1456	*α*-Humulene	KI and MS
31	19.63	0.13 ± 0.01	2.76 ± 0.05	1473	1472	*γ*-Gurjunene	KI and MS
32	19.85	0.18 ± 0.01	-----	1480	1480	Germacrene-D	KI and MS
33	20.77	0.37 ± 0.01	-----	1483	1484	*α*-Curcumene	KI and MS
34	21.21	0.51 ± 0.01	-----	1499	1500	*α*-Muurolene	KI and MS
35	21.78	1.49 ± 0.05	-----	1524	1525	*δ*-Cadinene	KI and MS
Oxygenated Sesquiterpenes							
36	20.95	1.54 ± 0.03	-----	1515	1514	Shyobunone	KI and MS
37	21.67	6.51 ± 0.07	2.31 ± 0.02	1517	1517	6-epi-Shyobunol	KI and MS
38	22.37	0.12 ± 0.01	-----	1518	1518	6-epi-Shyobunone	KI and MS
39	23.19	0.41 ± 0.01	-----	1563	1562	Citronellyl iso-valerate	KI and MS
40	23.47	3.33 ± 0.08	0.87 ± 0.01	1564	1564	*trans*-Nerolidol	KI and MS
41	23.66	7.67 ± 0.05	1.63 ± 0.04	1571	1572	Isoshyobunone	KI and MS
42	23.78	3.43 ± 0.04	30.86 ± 0.12	1575	1575	Spathulenol	KI and MS
43	24.13	0.17 ± 0.01	-----	1581	1582	Caryophyllene oxide	KI and MS
44	24.52	4.82 ± 0.09	0.95 ± 0.03	1584	1586	7-Hydroxyfarnesen	KI and MS
45	24.62	0.51 ± 0.01	1.25 ± 0.02	1595	1595	Salvial-4(14)-en-1-one	KI and MS
46	24.78	0.84 ± 0.02	-----	1596	1598	Veridiflorol	KI and MS
47	24.85	2.41 ± 0.05	-----	1608	1610	Humuladienone	KI and MS
48	24.97	0.55 ± 0.01	-----	1613	1613	Longifolenaldehyde	KI and MS
49	25.12	0.16 ± 0.01	-----	1625	1627	Isospathulenol	KI and MS
50	25.29	0.88 ± 0.04	-----	1621	1620	Fonenol	KI and MS
51	25.43	0.74 ± 0.03	-----	1641	1640	Cubenol	KI and MS
52	25.58	0.76 ± 0.02	3.65 ± 0.07	1642	1642	τ-Cadinol	KI and MS
53	25.65	0.38 ± 0.02	-----	1643	1644	τ-Muurolol	KI and MS
54	25.98	1.39 ± 0.06	-----	1649	1650	*β*-Eudesmol	KI and MS
55	26.88	0.29 ± 0.01	-----	1653	1654	*α*-Cadinol	KI and MS
56	27.04	1.74 ± 0.08	-----	1668	1668	Cedr-8-en-13-ol	KI and MS
57	27.5	0.40 ± 0.02	-----	1671	1670	Calarene epoxide	KI and MS
58	28.61	0.18 ± 0.01	-----	1682	1680	Ledene oxide-(I)	KI and MS
59	28.89	1.11 ± 0.03	6.34 ± 0.05	1683	1683	*α*-Bisabolol	KI and MS
60	30.66	-----	1.53 ± 0.03	1690	1693	6-Isopropenyl-4,8a-dimethyl-1,2,3,5,6,7,8,8a-octahydro-naphthalen-2-ol	KI and MS
61	31.41	-----	4.68 ± 0.07	2257	2259	4,4-Dimethyl-tetracyclo[6.3.2.0(2,5).0(1,8)]tridecan-9-ol	KI and MS
62	33.35	-----	0.96 ± 0.01	2462	2463	Isocalamendiol	KI and MS
Carotenoid Derived Compounds							
63	16.10	-----	0.97 ± 0.04	1279	1280	Vitispirane	KI and MS
64	16.45	-----	1.06 ± 0.04	1288	1287	Dihydroedulan II	KI and MS
65	23.29	0.64 ± 0.03	-----	1444	1445	Citronellyl propionate	KI and MS
Apocarotenoid Derived Compounds							
66	38.29	1.51	18.12 ± 0.11	1845	1845	Hexahydrofarnesyl acetone	KI and MS
Non-oxygenated Hydrocarbons							
67	32.32	-----	1.08 ± 0.06	1533	1535	2,6,10-Trimethyl-tetradecane	KI and MS
68	33.97	-----	0.97 ± 0.01	1885	1883	2,6,10,15-Tetramethyl-heptadecane	KI and MS
69	39.53	-----	1.57 ± 0.05	1900	1900	*n*-Nonadecane	KI and MS
70	44.39	-----	0.66 ± 0.04	2200	2200	*n*-Docosane	KI and MS
1	46.11	-----	1.05 ± 0.03	2300	2300	*n*-Tricosane	KI and MS
72	46.35	-----	1.28 ± 0.07	2500	2500	*n*-Pentacosane	KI and MS
73	52.20	-----	1.49 ± 0.05	2900	2900	*n*-Nonacosane	KI and MS
74	57.64	-----	0.42 ± 0.04	3000	3000	*n*-Triacontane	KI and MS
75	57.71	-----	0.46 ± 0.03	3200	3200	*n*-Dotriacontane	KI and MS
Oxygenated Hydrocarbons							
76	37.85	-----	3.73 ± 0.07	1942	1945	*cis*-9-Hexadecenoic acid	KI and MS
77	47.39	-----	0.29 ± 0.01	2135	2132	9,12-Octadecadienoic acid	KI and MS
78	47.42	-----	0.32 ± 0.01	2243	2246	9-hexyl-Heptadecane	KI and MS
	Total	98.55	99.64				

^a^ Rt: retention time; ^b^ values are mean (*n* = 2) ± SD of Saudi ecospecies; ^c^ Egyptian ecospecies; ^d^ literature Kovats retention index; ^e^ experimental Kovats retention index; MS: mass spectral data of compounds; KI: Kovats indices with those of Wiley Spectral Library collection and National 104 Institute of Standards and Technology (NIST) Library database.

## Data Availability

Not applicable.

## Sample Availability

Samples of the compounds are not available from the authors.
